# First Italian report of a liver abscess and metastatic endogenous endophthalmitis caused by ST-23 hypervirulent *Klebsiella pneumoniae* in an immunocompetent individual

**DOI:** 10.1007/s15010-022-01879-8

**Published:** 2022-07-08

**Authors:** Maria A. De Francesco, Giorgio Tiecco, Erika Scaltriti, Giorgio Piccinelli, Silvia Corbellini, Francesca Gurrieri, Verena Crosato, Giovanni Moioli, Valentina Marchese, Emanuele Focà, Davide A. Bertelli, Francesco Castelli, Arnaldo Caruso

**Affiliations:** 1 Institute of Microbiology, Department of Molecular and Translational Medicine, University of Brescia-ASST Spedali Civili, P. Le Spedali Civili, 1, 125123 Brescia, Italy; 2 Division of Infectious and Tropical Diseases, Department of Clinical and Experimental Sciences, University of Brescia and ASST Spedali Civili, Brescia, Italy; 3 Risk Analysis and Genomic Epidemiology Unit, Istituto Zooprofilattico Sperimentale Della Lombardia E Dell’Emilia Romagna, Parma, Italy

**Keywords:** HvKp, Invasive, Abscess, Liver, WGS

## Abstract

**Background:**

*Klebsiella pneumoniae* is a common species in the gut of mammals and is widely distributed in the environment. However, the environmental source of hvKp that precedes gut colonization is unclear, but once that it reaches the gut there is a possible generalized spread y fecal-oral transmission especially in endemic areas. Liver abscess might develop when the bacteria, using its virulence factors, cross the intestinal barrier and invade the liver by the portal circulation. This syndrome, prevalent mostly in Asian countries, is increasingly reported in Western Countries and leaves open questions about the source of infection.

**Case:**

Here we describe for the first time in Italy, a case of pyogenic liver abscess caused by a hypervirulent *Klebsiella pneumoniae* (HvKp) complicated by endophthalmitis and other metastatic infections in lung and prostate in an immunocompetent Chinese healthy individual with no recent travel in Asia.

**Conclusion:**

This case underlines the need for increased awareness of hypervirulent *K. pneumoniae*, even in settings where it occurs infrequently and where there are not evident epidemiological links.

## Introduction

*Klebsiella pneumoniae* is a Gram-negative bacterium responsible of community-acquired and healthcare-associated infections, such as urinary tract infections, pneumonia and bacteremia especially in immunocompromised patients [1]. Besides this classical strain, a hypervirulent *K. pneumoniae* (HvKp) strain has emerged, which is associated to severe community-acquired diseases in young healthy subjects without underlying pathologies [2, 3]. HvKp causes severe monomicrobial cryptogenic pyogenic liver abscess [4] and metastatic infections including endophtalmitis, bacteremia and brain abscess [5]. The invasive diseases caused by these strains are linked to chromosomal and plasmidic genes expressing specific virulence factors, a hypermucoviscous phenotype confirmed by a positive string test, a capsular serotype K1 or K2, a *rmpA* (regulator of mucoid phenotype) gene, a mucoviscosity associated gene A (*magA*) and the genes encoding aerobactin and its receptor [6, 7].

Generally, these strains are susceptible to different categories of antibiotics, except for an intrinsic resistance to ampicillin. However, the appearance of hypervirulent strains carrying multidrug resistance seems to be a consequence of an horizontal transfer of plasmid-mediated resistance [8].

HvKp strains belong principally to the clonal group CG23, which includes ST23, ST26, ST57 and ST163 and are prevalent mainly in Southeast Asia spreading then worldwide through Europe, Oceania, North America, South America and Africa.

Sporadic cases of liver abscess or metastatic infections due to HvKp have been reported from Europe [9–12].

Here we describe for the first time in Italy, a case of pyogenic liver abscess caused by a ST-23 HvKp strain and detected in an immunocompetent Chinese subject.

## Methods

Identification of the isolate was performed by VITEK-MS Matrix Assisted Laser Desorption/Ionization -Time of Flight Mass Spectometry (MALDI-TOF-MS; BioMérieux, Florence, Italy). Hypermucoviscous phenotype was confirmed by a positive string test. Briefly, it was performed by stretching a bacterial colony on a 5% sheep blood agar plate and was considered positive with a formation of a viscous string > 5 mm in length.

Antimicrobial susceptibility test was performed by automatic microdilution broth (VITEK®2 system, BioMérieux). Etest method (BioMérieux) wasused for imipenem, meropenem, ceftazidime/avibactam and ceftolozane/tazobactam, whereas the broth microdilution method was used for colistin as recommended recently by the European Committee on Antimicrobial Susceptibility Testing (EUCAST) and the Clinical and Laboratory Standards Institute (CLSI). The results were interpreted according to the 2021 EUCAST guidelines (https://eucast.org).

We sequenced the isolate (2021-097944-001-01) on a Nextseq platform (Illumina, San Diego, CA) using Illumina® DNA Prep (M) Tagmentation kit (Illumina, San Diego, CA) for library preparation. Reads were trimmed with Trimmomatic ver. 0.38 and assembled using Unicycler ver. 0.4.8. In silico Multi Locus Sequence Typing was determined using the Pasteur BIGSdb for *Klebsiella pneumoniae* (https://bigsdb.pasteur.fr/klebsiella/klebsiella.html) with Pasteur scheme. The online tool Pathogenwatch (https://pathogen.watch/) was used for analysis starting from the assembly that passed quality control with more than 95% of *Klebsiella* core genes detected. Antimicrobial resistance genes, virulence genes (as well as the derived virulence score), and capsule type genes were identified using Kleborate via-Pathogenwatch [13]. As a confirmation, ResFinder-3.2 was used to detect Antimicrobial Resistance Genes (https://cge.cbs.dtu.dk/services/ResFinder/), while the presence of plasmids detected by PlasmidFinder was checked manually by mapping of the raw reads.

A Minimum Spanning Tree (MST) based on cgMLST clustering was visualized using Microreact interface, choosing an allele threshold of 16 alleles. Core SNP analysis was performed with kSNP3 software (https://sourceforge.net/projects/ksnp/files/) starting from 127 assemblies of ST23 genomes selected from Microreact (allelic distance with 2021-097944-001-01 isolate > 16 alleles). A Maximum Likelihood (ML) phylogenetic tree was inferred starting from kSNP3 core SNPs matrix using PhyML with 100 bootstraps replicates.

Data are available at EBI under study accession n. PRJEB48936.

## Case

A 56-year-old Chinese patient was admitted to the Ophthalmology Unit of Spedali Civili’s Hospital, Brescia, Italy, for fever (up to 39.4 °C), right ocular pain, and a significant decrease of visual acuity. The patient had been living in Italy since 2004 and his last travel to China was in 2016. He worked as a tailor and denied risk factors for sexually transmitted diseases.

He had no relevant medical history of chronic diseases and reported no treatments except for sporadic use of inhaled corticosteroids for asthmatic symptoms. He claimed recent treatment with unspecified natural products for ocular pain, however, without any intravenous administration.

**Fig. 1:**
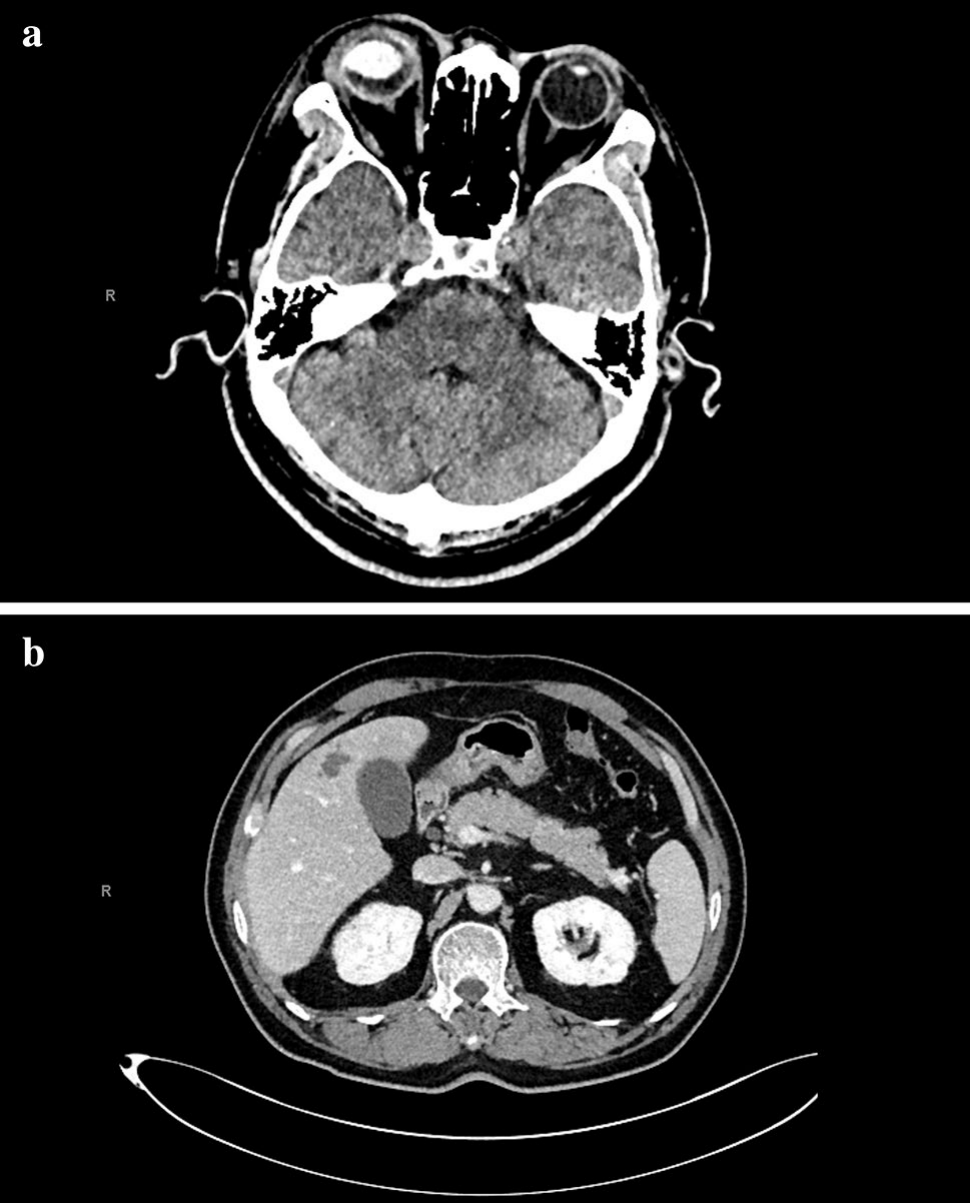
**a** CT scan of the Brain: diffuse thickening and blurred demarcation of right ocular walls. Exophthalmos associated with suffusion of glandular and periorbital adipose tissue. **b** CT scan of the Abdomen and Pelvis: hepatic non significantly vascularized lesion (25 × 14 mm) between 5 (V) and 8 (VIII) segments

An orbital computed tomography (CT) was performed, showing right ocular swelling with central enhancement, findings suggestive for endogenous endophtalmitis (Fig. 1a). On admission, laboratory tests revealed a white blood cell count of 11.29 × 10^3^/µl (reference value, 4.00–10.80); a relative neutrophilia (86.8%), a normocytic normochromic anemia (Hb 13.6 g/dl, MCV 90.3 fl) and a raised C-reactive protein value (243.3 mg/L). Renal, liver and electrolyte parameters were within normal ranges.

Empiric antimicrobial treatment was started with ceftriaxone 1 g IV every 12 h and vancomycin 500 mg IV every 12 h before Infectious Disease consultation. Blood and urine culture tests resulted negative. On day 4 after admission, the patient underwent right ocular vitrectomy with purulent discharge; cultural tests were performed with negative results.

Following Infectious Disease consultant’s prescription, a total body CT scan was performed revealing multiple abscesses (Fig. 1b): a 25 × 14 mm liver abscess between 5 (V) and 8 (VIII) segments (25 × 12 mm), a 9 mm (maximum diameter) upper right lung abscess and a 30 mm (maximum diameter) prostate abscess. Empiric antimicrobial treatment was switched and enhanced with piperacillin/tazobactam 4.5 g IV every 6 h, whereas vancomycin and ceftriaxone were discontinued.

On day 12 after admission, the patient was transferred to the Infectious Diseases Unit. Despite the presence of minimal respiratory symptoms, a sputum culture was performed and a pansensitive *K. pneumoniae* was isolated. Assuming an invasive multifocal Gram-negative infection, piperacillin/tazobactam was stopped in favor of ceftazidime 2 g IV every 8 h for a better ocular penetration. On day 14 after admission, the patient was apyretic and a hepatic needle aspiration with drainage of the abscess was performed. Cultures from hepatic abscess grew a hypermucoviscous *Klebsiella pneumoniae*.

Considering the clinical evolution and the microbiological result, a presumptive diagnosis of HvKp primary liver abscess with metastatic foci of infection was made.

Over the following weeks, the patient’s clinical conditions improved, with a decrease of inflammation markers: a white blood cell count of 4.91 × 10^3^/µl and a negative C-reactive protein (< 2.9 mg/L).

On day 41 after admission, antibiotic treatment was suspended and no relapse has been reported to date. He prosecuted ocular follow up, but unfortunately suffered from functional and probably irreversible blindness at the right eye. He was discharged after 46 days of hospitalization.

## Genomic results

The isolate, named 2021-097944-001-01, was assigned to the sequence type ST23 (*gapA-infB-mdh-pgi-phoE-rpoB-tonB* allele number 2-1-1-1-9-4-12), while PlasmidFinder via-Pathogenwatch tool identified the virulence factors-associated repB plasmid (KpVP-1, NTUH-K2044 plasmid pK2044, Acc.N. AP006726.1) [14] with an identity of 100%.

In particular, Kleborate analysis revealed that our isolate carried the “yersiniabactin (*ybt*) lineage *1*” genetic maker associated with the *K. pneumoniae* integrative conjugative element 1 (ICE*Kp3*) and a resulting yersiniabactin sequence type (YbST) 54. The colibactin lineage was clb 2 with a colibactin sequence type (CbST) 29-1 LV. The aerobactin lineage was *iuc 1* with an aerobactin sequence type (AbST) 1. The salmochelin lineage was *iro 1* with a salmochelin sequence type (SmST) 24-1LV 1. Capsule (K) and O antigen loci were KL1 (gene *wzi1*) and O1v2.

All these virulence determinants together with the virulence loci associated with hypermucoid phenotype (rmpA and rmpA2) were identified as located in pK2044 plasmid. In relation to these virulence factors, our isolate had been associated to Rm sequence type (RmST) 70. It also carried the *mrkABCDHIJK* and *fimABCDEFGHIK* genes encoding type 3 and 1 fimbriae, respectively, probably located on the chromosome.

The isolate carried only the *bla*_SHV-11_ β-lactamase gene conferring intrinsic resistance to the penicillins and supporting the suggestion that the isolate belongs to the HvKp pathotype. This also corresponded to the results of phenotypic AST, which revealed susceptibility against most of the antimicrobials tested including amoxicillin/clavulanate, extended spectrum cephalosporins, carbapenems, aminoglycosides, fluoroquinolones, rifampin and colistin.

**Fig. 2:**
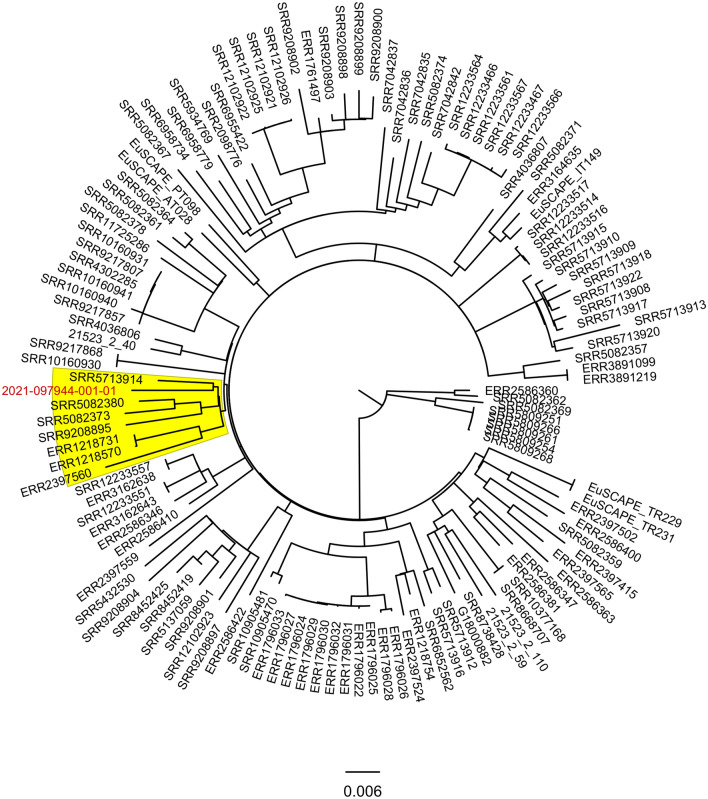
ML phylogenetic tree containing 2021-097944-001-01 isolate (in red) with 127 genomes of *Klebsiella pneumoniae* ST23 selected from Microreact (allelic distance with 2021-097944-001-01 isolate > 16 alleles). In yellow box the six isolates that are nearest to our isolate (same node) with a pairwise difference of maximum 575 SNPs with our isolate

Clustering analysis based on cgMLST revealed that 127 genomes of Klebsiella pneumoniae of ST23 had a maximum distance of 16 alleles from our isolate. A more detailed SNP analysis of these ST23 isolates with our *K.pneumoniae* isolate highlights that the nearest isolate (Isolate SB42; ID SRR571314), isolated in the Netherlands from blood, is away 138 SNPs from our isolate (highlighted in red in Fig. 2). Furthermore, the six isolates at the same node of our isolate differ from a maximum of 575 SNPs with our isolate (in yellow box in Fig. 2). Among these isolates, two of them (strains NUH01 and NUH02; ID SRR5082373 and SRR5082380, respectively) had been isolated from liver abscess infections in Singapore.

## Discussion

We report the case of a patient with invasive infection caused by an HvKp strain. The patient presented with a prolonged febrile illness and ocular pain. He had as only risk factor the Chinese origin, even if he had residing in Italy for 17 years without recent travels to China. HvKP infections frequently involve certain ethnic backgrounds (Asians, Pacific Islanders and Hispanics), rising speculative theories on predisposing genetic variation or increased frequency of colonization, especially in the setting of communitarian infections, as in our case.

HvKp is probably a member of the gut microbiome, which likely contributes to its diffusion in community setting. Previous studies showed that fecal carriage in healthy adults in China and other Asian countries was up to 75% [15].

Thus, this high prevalence of HvKP strains in Asian population might explain the high prevalence of invasive syndromes in this geographical area. Therefore, probably the strain colonized our patient’s gastrointestinal tract and the liver abscess might occur when the bacteria cross the intestinal barrier.

Despite these hypervirulent strains can affect immunocompetent subjects, as in our clinical case, invasive infection occurs predominantly in people with diabetes mellitus, getting severe and difficult-to-treat manifestations as endophtalmitis [16]. Prognosis of endophtalmitis is generally poor, with 89% of patients reporting partial or complete vision loss and an early diagnosis and treatment are important to improve clinical outcome.

Other studies reported an association between incidence of liver abscess and the use of some antibiotics such as amoxicillin and ampicillin within 30 days [17].

Then, in a large multicentric study, long-term systemic steroid was found associated to ESBL-producing HvKP strains, rather than to the increased detection of overall HvKP infection [18]. However, apart from the sporadic steroid administration, our patient did not report any significant drug exposure.

Hypervirulent *K. pneumoniae* strains isolated in Europe are generally community-acquired and susceptible to antibiotics [19]. However, even if the patient was treated with aggressive and appropriate antibiotic therapy, he suffered from functional blindness. On the contrary, reports from Asia describe an increase in strains carrying both resistance and virulence plasmids and for this clinical challenge, ECDC raises the alarm for a careful surveillance to prevent the emergence of these multidrug resistant strains [20]. As these strains are commonly hospital-acquired, multiple nosocomial outbreaks of ventilator-associated pneumonia due to carbapenemase-producing HvKp have now been reported across Asia, with uniformly negative outcomes [21]. This recently recognized development has blurred the traditional epidemiological statement that HvKp infection are community acquired and rarely resistant to antibiotics creating new challenges for both the clinician and microbiologists.

So far, our report underlines the need for clinicians to have an increased clinical suspicion about this pathogen, even in the absence of recent or previous travels to Asia, which may allow a better diagnostic and therapeutic management of patients with pyogenic liver abscesses without underlying causes.
